# Lifestyle Dimensions of Public Safety Personnel Families: There’s No Life Like It

**DOI:** 10.1007/s10926-024-10213-y

**Published:** 2024-06-13

**Authors:** Heidi Cramm, Marilyn Cox, Deborah Norris, Nathalie Reid, Linna Tam-Seto, Rachel Dekel, Nicola T. Fear, Lisa Delaney, Rachel Richmond, Alyson Mahar

**Affiliations:** 1 School of Rehabilitation Therapy, Queens University, Kingston, ON Canada; 2 Department of Family Studies and Gerontology, Mount Saint Vincent University, Halifax, NS Canada; 3 Child Trauma Research Center, University of Regina, Regina, SK Canada; 4 Department of Occupational Science & Occupational Therapy, University of Toronto, Toronto, ON Canada; 5 School of Social Work, Bar-Ilan University, Ramat Gan, Israel; 6 King’s Centre for Military Health Research, King’s College London, London, UK; 7 School of Nursing, Queen’s University, Kingston, ON Canada

**Keywords:** Public safety families, Work-family conflict, Family resilience, Family well-being, Risk factors, Role overload, Family dynamics

## Abstract

**Purpose:**

The nature and cumulative occupational demands imposed on families of public safety personnel (PSP) are substantial, in many cases non-negotiable, and distinct from the general population accentuating risk factors for family well-being. Despite this reality, the contributions of PSP families are not well understood, and a conceptual framework is needed. The aim of this paper is to summarize contextual factors (lifestyle dimensions) that shape the lives of PSP families; factors supported in the existing, albeit limited, body of research.

**Methods:**

Grounded in the interpretive/constructivist paradigm, a synthesis was central to understanding the lived experiences of PSP families. An interdisciplinary research team engaged in an iterative process of framework analysis to capture the variability and complexity of PSP family life and distilled the overarching lifestyle dimensions.

**Results:**

Three lifestyle dimensions—logistics, risks, and identities—emerged from contextual factors and represent distinct aspects of PSP family life. PSP families play a crucial role in that their capacity to accommodate the lifestyle dimensions (i.e., logistics, risks, and identities), without which the PSP could not meet the demands of the profession.

**Conclusion:**

Promoting awareness of these dimensions and their consequent demands underscores the cumulative demands that put PSP families at risk. Responses from governments, public safety organizations, and communities are required to help PSP families manage non-negotiable elements of the public safety occupation that spill over into family life over which they have no control.

## Introduction

Public safety personnel (PSP) (e.g., firefighters, paramedics, correctional workers) provide essential emergency services 24/7 to their communities and the nature of their work continuously exposes them to potentially psychologically traumatic events (PPTE) which have been associated with mental health issues such as posttraumatic stress injuries (PTSI), depression, and general anxiety disorder [1, 2]. In addition, shiftwork, long hours, and organizational pressures such as workload and a military-like chain of command can influence sleep quantity and quality, affect memory and mood, and increase the risk of chronic disease [3–5]. These factors inevitably spill over into home life; PSP families are impacted as PSP try to balance a potential pile up of stressors associated with the risks and requirements of the job with their roles and responsibilities at home [6]. The requirements of public safety occupations put demands not only on PSP but also on families who must adapt to a way of life which is expected to accommodate the job commitments of PSP, nonstandard schedules, and the hazards that PSP are subject to in their work environments.

The influence and vulnerability of families to outside forces is an element of applied research focused on family well-being. The functions and relationships of individual family members outside the family system (e.g., job, school, social networks) influence interactions within the family system [7]. The significant demands of public safety work impact family relationships just as family life can enhance or impair the ability of PSP to perform in the workplace. Public safety organizations rely on the commitment of PSP and the support of PSP families to fulfill their mandate to provide around the clock emergency response. We argue that PSP families represent a distinct group and there is a need to recognize and support the families who serve alongside essential workers.

The aim of this paper is to summarize lifestyle dimensions (i.e., contextual factors) that shape the lives of PSP families. A comprehensive understanding of these lifestyle dimensions can be used to highlight the strengths and vulnerabilities of PSP families, enhance evidence-informed programming and policy, and identify gaps in the research. This paper begins with background on concepts, the limitations of existing literature on PSP families, and the origins of the contextual factors from which a body of literature has evolved for military families. The process which elicited the lifestyle dimensions will be outlined and discussed followed by implications for research, policy, and programming.

## Background

### PSP Family

In accordance with Public Safety Canada, PSP are defined broadly and “meant to encompass front-line personnel who ensure the safety and security of Canadians” [8]. They “include but are not limited to, border services officers, public safety communicators, correctional workers, firefighters (career and volunteer), Indigenous emergency managers, operational intelligence personnel, paramedics, police (municipal, provincial, federal), and search and rescue personnel” [9, para. 4]. There are many interpretations of what constitutes family, and we subscribe to a subjective definition aligned with the lived realities of people who call themselves “family.” The concept of family is dependent on culture and purpose [10] and is applied within the context of the PSP occupation being sensitive to both internal (family values and beliefs) and external forces (occupational risks and requirements) that shape relationships within the family system. A subjective and inclusive definition of family extends the reach of applied research [11] and, therefore, we describe families as self-determined systems formed through interpersonal relationships characterized by emotional, physical, and social bonds [12]. The structure, function, and physical and social realities within the family system are complex and change over the life course of an individual, as well as the life cycle of the family [13]. Families are both a unit of study in and of themselves, and family processes affect the growth, conduct, and welfare of individuals [14]. The career stage of the PSP (e.g., new recruit, retiree) and the developmental stage of the family are important variables. For example, young families of newly recruited PSP experience different challenges than either families of PSP in mid-career with children in college or families of PSP near the end of their careers with adult children. We focus on the impacts of the public safety occupation on the family recognizing that the effects are bidirectional, and an individual family member’s job performance is likewise influenced by family processes (i.e., work-to-family conflict and family-to-work conflict).

### Lifestyle Dimensions

The use of the concept of *lifestyle* also requires clarification. PSP families must adapt a *lifestyle* to accommodate the demands of the PSP occupation that pervade their everyday lives. As Jensen [15] points out, *lifestyle* is interpreted differently depending on the discipline and can relate to overall health (e.g., habits, diet), what is consumed (e.g., brands), or how an individual or group goes about life, which is the definition applied here. In the context of this research, we use *lifestyle dimensions* to describe the contextual factors that are typical, common, or representative of PSP families. How these lifestyle dimensions impact, and are impacted by, individual and community culture, health and well-being, education, income, geographical location, and occupation is pertinent to this discussion.

### Research on PSP Families

Recent reviews have shown that a body of literature on PSP families has been slow to emerge and is segmented [16–18]. Cox et al. [16] showed that many sectors of public safety such as communications (e.g., emergency services dispatchers), paramedics, and corrections have been neglected and that primary research favors the PSP, excluding the perspectives of other family members. A preliminary review of the literature by Leroux et al. [17] concurs with these findings and adds that existing research on PSP families focuses primarily on the health and well-being of PSP rather than the effects on other family members and their experiences. Sharpe et al.’s [18] systematic review on the mental health of PSP (emergency responder) families found most studies were situated in the USA, targeted police families, garnered data only from the PSP family member, and focused on the impacts on the PSP. It is evident that we have much to learn about PSP families from different public safety sectors with a focus on the effects of PSP occupations on the family and told from the perspective of all family members.

### Military Families

There are parallels between PSP families and military families due to a similarity in the demands that these occupations pose (e.g., potential exposure to trauma, unpredictability) [19]. Though there are also dissimilarities, the factors identified in the military family literature are useful in advancing our understanding of PSP families. Drummet et al. [20] summarized three overarching contextual factors that influence military families: relocation, separation, and reunion related to the transient nature of military service and deployment cycles. Additionally, the National Defense and Canadian Armed Forces Ombudsman [21] drew on a landmark study on “greedy institutions” [22], describing the contextual factors (characteristics) of military life as mobility, separation, and risk. The risk of physical injury, illness, or death and operational stress injuries, along with disruptions related to relocation and lengthy absences by a military family member, imposes significant demands on military families [23]. It is this intersection of the military and family that is of interest. As Segal [22] noted, “both make great demands of individuals in terms of commitments, loyalty, time, and energy” [22, p. 9].

In contrast to the lack of family-centered literature on PSP [16–18], a scoping review by Manser [24] showed that a well-developed body of literature on military families has emerged over the past decade which has been proposed as a frame of reference for PSP families [19]. Similar to military families, PSP families must accommodate occupational risks and requirements, and research has shown that these factors heighten demands [25–27], necessitating appropriate evidence-based resources to help mitigate negative outcomes.

Using the military family literature as a frame of reference requires that we also focus attention on the dissimilarities. For example, PSP families need to manage the exit and reentry of PSP daily [28]; PSP typically work rotating and sometimes long shifts facing adversity and risk in their home communities, returning to their families within a 24-h period [29, 30]. In contrast, military personnel often find themselves in combat in other countries and deployments are months or years [31]. These differences, among others, set PSP families apart from military families which underscores the need for dedicated research on the distinctive nature of PSP family life. The body of literature on military families serves as a guide for emerging research on PSP families with the caveat that relying too much on the military family literature diminishes and neglects the unique experiences of PSP families. The nuanced context of PSP families must be understood and accounted for.

### Family Resilience

Advancing research on PSP families and filling the gaps requires that we lay the groundwork by identifying those aspects of everyday life that are unique while simultaneously acknowledging the complexity and variability among and between different PSP sectors and contemporary family structures. There is evidence that the impact of normative family stressors on family well-being is compounded by the occupational risks and requirements of public safety work [16, 18]. Many of the occupational challenges are not exclusive to PSP families (e.g., shiftwork and overtime); however, the combination and magnitude of the demands heightens risk factors for negative mental health outcomes.

Patterson’s [32] family adjustment and adaptation response (FAAR) model, applied extensively in military family literature [33–35], illustrates the vulnerability of families when a pile up of both normative and nonnormative demands occurs. The FAAR model focuses on the family system and proposes that family capabilities (i.e., resources and coping behaviors) must be sufficient to balance demands to avert a crisis. This model is useful in understanding the salience of contextual factors for PSP families. In the face of the normative demands (e.g., the anticipated birth of a child, and death of an elderly family member), family strengths and resources may be sufficient, and the family is able to adjust (short-term change) and/or adapt (long-term change). However, when these normative demands are accompanied by nonnormative demands (e.g., injury), they may exceed family capabilities. As Segal [22] noted regarding military families, it is how occupations and family life intersect that is of particular interest. The family might struggle to care for the newborn even though they prepared for the baby's arrival due to the co-occurrence of unanticipated events. It is both the cumulative effect and the magnitude of events that challenge the capabilities of families [32].

## Methods

Grounded in the interpretive/constructivist paradigm [36], we explored the intersection of family life and public safety occupations to generate the lifestyle dimensions which are central to our understanding of PSP families. Evidence of the lived experience of this population was interpreted by the authors “to offer the inquirer’s construction of the constructions” [36, p. 222]. Due to the noted absence of family voices and the limited research on PSP families, this “construction” is foundational for a body of research in its infancy, yet contingent on the first voice accounts of future research. The research team (see authors) included researchers, associates, and trainees from disciplines within health science, family science, and military and veteran health who met weekly over a 2 month period via videoconferencing. Using the a priori contextual framework (i.e., mobility, separation, and risk) developed by military scholars [20, 22], the research team discussed the contexts and contingencies associated with the lived experiences of PSP families engaged in intersubjective, co-created practices instrumental in developing and maintaining resiliency. The findings and interpretations of authors from three recent peer-reviewed systematic literature reviews [17, 18, 37] and one narrative review [16] were interwoven with the military contextual framework to form the basis of this investigation. New understandings and reconstructions were derived through reflexive discourse and constant comparative analysis [38].

Framework analysis, which supports both inductive and deductive exploration [39], was adopted to address the contextual concepts. Designed to be dynamic, systematic, and comprehensive [40], framework analysis facilitated a non-linear, reflexive, and iterative process. We aligned our research with the five key stages of this method which included familiarization, identifying the thematic framework, indexing, charting, and mapping [40].

*Familiarization* involved consultation and a collection of key findings. The research team compared the emerging research on PSP families with the more advanced body of literature on military families focused on contextual factors*. Identifying a thematic framework* involved a process of collaboration whereby multiple themes were presented and discussed to clarify meaning, importance, and interrelatedness. Concepts were introduced to integrate common themes and, through a process of renaming, collapsing, and reordering, a preliminary framework emerged to reflect the data. The vast number of sub-themes was *indexed*, and diverging opinions regarding categorization were reconciled. The *charting* stage required the research team to condense and reorganize the findings to focus on interrelationships. As the research team shifted from indexing to charting, the concepts were refined to ensure that interpretations were accurately reflected in the labels. Charting was done using spreadsheets and a conceptual map created in MAXMaps [41] illustrating the scope and interconnectedness of concepts.

*Mapping*, the final stage of framework analysis, involved defining key concepts and further reflection to ensure that the range of PSP family experiences were captured. When consensus was reached, the research team engaged in a synchronous writing exercise to develop narratives depicting the complexity and variability of PSP families. The intent of the simulation was to test the adequacy of the contextual framework to reflect the experiences of this population. PSP family members (also authors/researchers on the team) provided feedback on the narratives. Systematically moving through the five stages of framework analysis provided transparency of process and an audit trail which can be revisited. The integration of new information and feedback, essential for an emerging field of inquiry, is intrinsic to the process.

## Synthesis

**Fig. 1:**
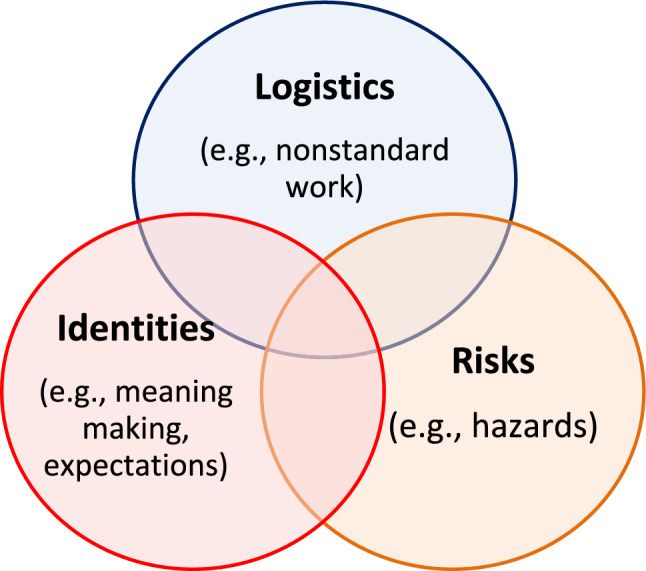
Synthesis of the Lifestyle Dimensions of PSP Families

Like all families, PSP families experience a dynamic set of challenges as they balance the demands and needs of each family member with the requirements of work. However, the heightened risks and requirements associated with public safety work create opportunities and challenges which differentiate PSP families and shape the lifestyle. PSP families experience a unique convergence of transacting lifestyle dimensions that intensify and magnify regular family stressors. Multiple themes and sub-themes which emerged from our analysis and mapping process culminated in three overarching lifestyle dimensions common to PSP families: *logistics*, *risks*, and *identities*. Each dimension focuses on distinct aspects of PSP family life influenced by the public safety occupation, and together have overlapping and cumulative effects. These dimensions also change in their influence and weighting at different points in a family’s life cycle and in the PSP’s work and occupational exposure trajectory. Figure 1 illustrates the interrelatedness of the dimensions and the descriptions that follow capture the range of themes and sub-themes that are interwoven in each dimension.

### Logistics

Logistics captures structural forces of PSP work, like shiftwork, that shape day to day family life. PSP families are faced with changing PSP work demands that disrupt family schedules and routines, introducing tensions and work-family conflict within the family system. Depending on the family life stage, some families are able to capitalize on the nonstandard work schedules of PSP to respond to family care needs and requirements through a coordinated tag team approach [42, 43]. A hallmark feature of this dimension is the *microtransition* [44], where many PSP shift frequently and abruptly between family life and work. Shift changes, call-ins, and overruns make the entry and exit of the PSP family member uncertain and potentially difficult. Families are not always sure how to engage and communicate during these transitions [45]. Families sometimes also experience a sense of being out of sync with the broader community particularly during widely celebrated holidays when many PSP are on shift, engendering a sense of loneliness and isolation [46]. Community involvement and connections with extended family are limited by the nonstandard schedules and unpredictability of PSP work. The logistics require families to adapt to different ways of being which often depart from the norm [47, 48].

Structural forces affect things like sleep, childcare, couple activities, intimacy, coparenting, shared meals, family time, household management, participation in ongoing extracurriculars, as well as special events, and, for some, relocation [16]. The impacts are compounded when a spouse or significant other also works nonstandard hours [49]. The dynamic structural forces on families sometimes impact the spouse or significant other’s career choices, as they opt for more flexible work arrangements to accommodate operational imperatives of public safety work [26]. These forces create disproportionate family responsibilities and, depending on the family’s composition, potentially entrench traditional gender roles constraining women’s choices [3, 6] and careers. Depending on the sector, location, and career/volunteer nature of the PSP job, scheduling demands have different levels of predictability and precarity. These logistical issues are outside the family’s control and create both pressures and opportunities for families as they constantly plan, adapt, compromise, balance, manage, negotiate, coordinate, and optimize their responses to these non-negotiable structural forces [26, 46, 47, 50].

### Risks

The ways in which the physical and psychoemotional risks and hazards to which PSP are exposed through the course of their duties play out within the family system is summed up in the lifestyle dimension, *risks*. Risks and hazards can be exacerbated by organizational pressures such as staff shortages, solitary deployment to the field, shift overruns, or an inability to share confidential details about work which put further strain on families [47, 48].

Family members have a sense of the traumatic events PSP are potentially exposed to, informed by television, news, and movies [51, 52]. When emergencies occur in public view, social media coverage and graphic real-time images of hazardous situations intrusively put risk into view, intensifying fears and anxiety for PSP families [46, 53]. While family members are not typically exposed to the risks and hazards directly, they are often affected by the consequences for the exposed PSP through changes in behavior, communication, and relationships, which can lead to confusion, frustration, internalization, conflict, fear, and anxiety for non-exposed family members [3, 18, 54–56].

PSP exposure to trauma can result in emotional fatigue and mental health challenges [2] which require recovery making transitions between work and home challenging. PSP behaviors such as irritability, anger, and reactivity result from stress and unprocessed trauma, which negatively affect families [26, 52]. PSP sometimes withdraw from interactions with family and friends to cope with work stress; they are physically present but emotionally disengaged [5, 25, 46, 57]. This causes uncertainty in family relationships, diminishing mutual support and potentially resulting in suspicion and distrust [6, 26]. Self-medication with alcohol or drugs and infidelity are further threats to relationships [58, 59].

The military-like nature of public safety work often reinforces authoritarian behaviors that, when extended into family communication and role negotiation, create tension and conflict at home [60–62]. Hypervigilance is adaptive for PSP in their work roles; however, when that behavior transfers to the home environment, PSP who are overprotective and hypervigilant about risk, constrain family activities [18, 51]. These behaviors not only impact relationships but cross over to affect the well-being of other family members and, in some cases, put family members at risk of secondary trauma [56, 63]. Dual serving PSP couples who have a shared understanding of the nature of risks and hazards at work have reported less discomfort and more open communication [27, 64, 65], while non-PSP family members are at heightened risk of vicarious trauma through communication patterns focused on sharing potentially traumatic events [26, 52].

### Identities

PSP families occupy a unique place in the community, having a social identity and expectations conferred upon them by virtue of their association with a PSP family member. Through membership or association with an occupational group, the formation of a social identity may be personally enacted and accepted or dismissed and rejected. PSP families may label themselves differently at various points in the family life cycle, with some describing themselves as ‘a police family’ or ‘a fire family’ and others electing to distance themselves from that identity [46, 54]. For some, affiliation with and attachment to a particular PSP sector has been identified as a source of pride, belonging, and meaning [45, 52, 66]. Despite this connection, families do not always feel that their contributions are sufficiently recognized and appreciated by PSP organizations or the community at large [63, 67]. Other families see themselves as separate from the career or have different identities at the forefront of their family unit, such as religious, ethnic, or regional affiliations [45, 54]. Cultural factors amplify or complicate identities further [68], with the potential for different perspectives on what it means to be a PSP family and how cultural identity is preserved within those roles.

Families in various regions and PSP sectors gain social capital, described by [69] “as a sum of resources (symbols, opportunities, information, and supports) for the community that emanate from reciprocal relationships” [69, p.12], due to their association with a given sector [57]. Along with the benefits of belonging and having access to a particular social network, PSP families are also subject to the expectations of public safety organizations and communities [45, 54, 70]. The values and prioritization of the public safety organization sometimes conflict with family values, creating tension within families who compete with the PSP organization for primary status [45]. When the PSP work becomes a dominant element of their identity and sense of self, this tension can be magnified. For dual-career PSP households, the variable ways in which each spouse or significant other enacts the PSP identity as a ‘job’ or as ‘life’ further complicate family relationships [71].

Some members of the community consider families to be extensions of the PSP with expectations of accountability for and knowledge about PSP sector activities [51, 54, 72]. The challenges that PSP families face in the community and the demands imposed by public safety organizations on families are not well understood by the public. PSP families experience an outpouring of support and gratitude from the community when public perceptions are positive but also find themselves subject to criticism, especially when there have been negative media reports about a particular sector of public safety [51, 73].

## Discussion

### Embracing the Mess

The process of identifying and synthesizing lifestyle dimensions unique to PSP families was fraught with multiplicity, complexity, overlaps, and gaps. Consideration was given to (a) internal characteristics (emotional, psychological, embodied); (b) external characteristics (shiftwork, organizational requirements, community expectations); (c) historical characteristics (expectations, stoicism, military-like orientations); and (d) contexts (social, political, economic, geographical, cultural, religious, familial, organizational, etc.) in which PSP and PSP families are situated. The patterns of characteristics emerged over time and through reflexive discourse, requiring the research team to *embrace the mess* and engage in multiple iterations.

PSP families are inextricably involved in the public safety occupation alongside not only the PSP, but also alongside the organizations and communities in which they are situated. Multi-layered, multi-expectation, complex and continuous engagements with lifestyle dimensions are inherent to being connected with PSP work. While each PSP family lifestyle dimension is identifiable and distinct, overlapping realities also exist where each dimension shapes and is shaped by the others. Embracing and engaging in the mess required an acknowledgement of the multiple variables that influence the lives of PSP families.

### Interconnected Variables

The mapping process provided a visual cue of the complexity, revealing an intricate web of the interconnected variables. The simulation of PSP families developed through the synchronous writing exercise provided a richer understanding of how the public safety occupation intersects with family life. Family structure, life cycle, history, culture, gender, and geographic location, combined with the variability of lifestyle dimensions based on sector and career life cycle, make the experiences of each PSP family unique demonstrating the need for tailored resources and support.

An inclusive definition of *family* recognizes both biological and chosen family relationships, acknowledges the roles of all individuals who identify as “family,” and highlights the need to consider the impact of different family structures in the context of public safety occupations. The family system changes as individuals within it mature and experience normative events such as the addition of children, aging parents, separation, divorce, blended families, illness, and death. These changes, though frequently anticipated, can present challenges and disrupt family processes [32, 74], adding to the pile up of stressors associated with the risks and requirements of the PSP occupation. PSP families who have had little exposure to the lifestyle must simultaneously manage the normative challenges of family life and adjust to accommodate occupational demands, while families who include multiple generations of PSP may benefit from experience. Past experiences, unrelated to current circumstances, such as childhood traumas (e.g., childhood abuse, poor physical or mental health) also have an impact on family relationships [3] affecting behaviors which contribute to cumulative demands.

Gendered expectations in PSP families, where women are pressured to take on unpaid work related to household and caregiving responsibilities are prevalent in opposite-sex couples where the male partner works in male dominated public safety occupations such as policing [6, 75]. Ethnicity also plays a role in shaping family values and beliefs [76], which can impact perceptions of both family and public safety work. Cultures that prioritize family roles and rituals may conflict with nonstandard work schedules [77, 78], while family values aligned with a commitment to public safety work are advantageous [70].

The expectations of PSP families in the community are often influenced by the region in which they reside. Smaller communities have higher expectations and less anonymity [72], while large urban centers have a higher volume of critical incidents and crime [3]. Location can impact job advancement, organizational support, household income, and family support [71]. There are also variations across sectors due to differences in occupational risks, organizational expectations, and public perceptions [51, 70, 79]. The sector (e.g., paramedic, police, firefighter), motivation, ambition, and opportunities for advancement [80], and family life [48] influence career paths. Career stages across sectors can influence the demands on the family with early career PSP focused on acquiring skills and experience, while mid- and late-career PSP often have increased responsibilities associated with advancement [6].

## Implications

PSP families are not homogeneous and lifestyle dimensions are not intended to be rigidly defined; rather, they are modifiable to fit with sectoral and/or individual family circumstances. For example, our research showed that logistics impacts all PSP families and relocation is an aspect of logistics, but relocation only impacts certain PSP within specific public safety sectors (e.g., police) where mobility is required with postings to new locations. The advantage of the broader *dimensions* is that they are more inclusive of different public safety sectors and different experiences. Moreover, the lifestyle dimensions ascribed to PSP families that have been modified from contextual factors applied to military families [20–22] can be further modified and applied to other occupational sectors (e.g., healthcare workers, seafarers). Recognizing the overarching dimensions and the underlying contextual factors related to the public safety occupation allows a more comprehensive understanding of PSP families to emerge.

Research, theory, and practice draws on the lived, co-created experiences of the PSP family members as starting points. Emphasis on the *practices* undertaken by family members brings these points into view. Practices operate interdependently at individual, familial, community, and organizational levels. To what extent these practices are understood and responded to at each level determines the adequacy of resources and support to manage adversity which influences family resilience [14]. Emphasis on the visibility of PSP families and recognition of the challenges they face by public safety organizations, communities, and the public validates their experience and is an impetus for developing policy and programming.

Explicit focus on the diversity of families in terms of their form and function ensures that policy and programming do not perpetuate a narrow view of what “family” means. Families themselves matter, in their own right, and require services and support that respond directly to their needs. Family members serve alongside PSP and public safety organizations, meriting both recognition and support. This begins with an acknowledgement of their contributions and the demands imposed on them. An awareness by public safety organizations, communities, and the public that the occupational risks and requirements place nonnormative demands on PSP families is needed to direct applied research and tailor resources to enhance family capabilities.

Further research, particularly with those families in sectors of public safety work that have been understudied, is needed to refine the conceptualization of the lifestyle dimensions. A better understanding of life course experiences and the needs of PSP families as the PSP moves through their occupational trajectory will help target support. Prime examples would include studies focused on different family structures (e.g., blended families, single parents), different stages within the life course (e.g., young families, couples transitioning to retirement), and comparing the perspectives of the PSP with that of their family members. Consultations with PSP families will direct the prioritization and development of acceptable, accessible, evidence-based, and timely resources.

By identifying and naming the unique lifestyle dimensions of PSP families, we have created an affirming starting point for a population who may not have recognized all the structural forces at play in shaping their day to day lives. An application of the lifestyle dimensions developed in consultation with PSP families is currently being tested with the launch of an online psychoeducational resource hub for PSP families (https://www.pspnet.ca/en/for-families-of-psp). This is one outcome of this research with a further response needed from multiple levels including public safety organizations, communities, and the public. This synthesis offers a critical advance in the foundation for more targeted research and evidence-based guidance for programs and policies. A fulsome response will require partnership and collaboration between PSP families and those organizations and associations that serve and support them.

## Conclusion

We cannot begin to address the complex issues that influence the health and well-being of PSP families without an appreciation of their lived experience. Identifying the points at which family life and the public safety occupation intersect alerts us to both the demands and responses (adaptive and maladaptive). It is the family in context which will determine the relevance and effectiveness of PSP family research and practice.

The lifestyle dimensions presented here are foundational to advance research in this emerging field of inquiry. The contextual factors accounted for in the lifestyle dimensions and the variability of PSP families described address many of the complex challenges associated with this population. Prioritizing the first voice accounts of PSP families in future research will enhance these understandings. The three dimensions—logistics, risks, and identities—serve as an anchor identifying the cumulative elements that differentiate PSP families from the general population.

## Data Availability

The datasets analyzed during the current study are available from the corresponding author.
